# The association between the deviation from balanced time perspective on adolescent pandemic mobile phone addiction: the moderating role of self-control and the mediating role of psychological distress

**DOI:** 10.3389/fpsyg.2023.1298256

**Published:** 2024-02-08

**Authors:** Hao Zhang, Canjie Chen, Luoyi Zhang, Shuang Xue, Wanjie Tang

**Affiliations:** ^1^School of Education and Psychology, Chengdu Normal University, Chengdu, China; ^2^Guangzhou Panyu Polytechnic, Guangzhou, China; ^3^Faculty of Humanities and Social Sciences, City University of Macau, Macao, Macao SAR, China; ^4^Institute of Analytical Psychology, City University of Macau, Macao, Macao SAR, China; ^5^Department of Sociology and Psychology, School of Public Administration, Sichuan University, Chengdu, China; ^6^Mental Health Centre, Sichuan University, Chengdu, China

**Keywords:** adolescent mobile phone addiction, moderated and mediation analysis, balanced time perspective, self-control, depression and anxiety

## Abstract

**Background:**

Few studies have examined the impact that the deviation from balanced time perspective (DBTP) had on mobile phone addiction during the COVID-19 normalization prevention and control phase. Therefore, this study sought to determine the associations between DBTP, depression and anxiety, self-control, and adolescent mobile phone addiction.

**Methods:**

The moderated mediating model was tested using the SPSS PROCESS model. The sample was 1,164 adolescents from different regional areas of Sichuan, China. From February to March 2020, participants completed the Zimbardo time perspective inventory (ZTPI), the brief symptom inventory for physical and mental health (BSI-18), the self-control scale (SCS), and the Chinese version of the mobile phone addiction index (MPAI).

**Results:**

The DBTP was significantly and positively correlated with mobile phone addiction, depressive and anxiety symptoms mediated the relationship between DBTP and mobile phone addiction, self-control moderated the indirect effect of DBTP on mobile phone addiction, and as the level of self-control increased, the effect of DBTP on anxiety and depression and the effect of depression and anxiety on mobile phone addiction weakened.

**Conclusion:**

During the COVID-19 pandemic, DBTP and lower self-control were risk factors for higher mobile phone addiction in adolescents. Therefore, guiding adolescents to balance their time perspective and enhance their self-control could strengthen their psychological well-being and reduce addictive mobile phone behaviors. This research was supported by “Youth Fund of the Ministry of Education” (18YJCZH233): “Research on the plastic mechanism of decision-making impulsiveness of anxious groups in the context of risk society.”

## 1 Introduction

Marciano et al.’s (2022) meta-analysis concluded that prolonged isolation during the COVID-19 pandemic resulted in a significant reduction in adolescent offline social activities and an increase in the use of digital platforms and internet-based technologies for social contact, entertainment, and information sharing. This lack of meaningful face-to-face interactions has been found to have a detrimental impact on physical and mental development as evidenced by the significant increase in adolescent behavioral problems such as mobile phone addiction (Sun et al., 2020; Serra et al., 2021; Zhan et al., 2021). For example, Sun et al. (2020) found that 46.8% of participants reported a significant increase in smartphone dependence during the pandemic.

Smartphone addiction, a behavioral addiction, is characterized by an excessive addiction to mobile-mediated activities and a strong and persistent desire to use the mobile phone, which can lead to significant social and psychological impairments (Pradeep et al., 2022). Sicheng et al.’s (2021) meta-analysis found that the overall global prevalence of mobile phone addiction in both adults and adolescents was 28.3%. Previous research has shown that mobile phone addiction can have a significant negative impact on adolescent psychological development, with mobile phone misuse causing adverse psychological effects, sleep disturbances, (Zhang et al., 2022) poor academic performances (Grant et al., 2019), loneliness (Grant et al., 2019), anxiety (Yang et al., 2019) and depression (Park et al., 2019; Yang et al., 2019). It has been speculated that mobile phone addiction could become a major non-drug addiction in the 21st century, especially for adolescents (Yu and Wang, 2020; Pradeep et al., 2022). Therefore, reducing adolescent mobile phone addiction prevalence has become a key research focus.

Zimbardo’s time-theoretic perspective could assist in understanding the adolescent mobile phone addiction influencing factors as adolescents with no deviations from balanced time perspectives (DBTP) are less likely to have mobile phone addictions (Park et al., 2014). The cognitive-behavioral model of pathological internet use (Davis, 2001) and the dual systems model of self-control (Hofmann et al., 2009) identify two variables influencing adolescent mobile phone addiction; negative emotions and poor self-control. This study examined the potential adolescent mobile phone addiction influencing mechanisms during the COVID-19 pandemic to identify possible interventions to reduce adolescent mobile phone addiction prevalence.

### 1.1 Deviation from balanced time perspective and mobile phone addiction

The temporal perspective is a process that involves assigning temporal categories or time frames to personal and social experiences to provide coherence, meaning, and order to these experiences (Zimbardo and Boyd, 2015). Human experiences are divided into past, present, and future temporal categories, which are then used to encode, store, and recall experienced events. Most people have five different temporal cognitive tendencies; past positive, past pessimistic, present hedonistic, present fatalistic, and future-oriented; each of which influences perception shaping, expectation formation, direct attention, the provision of explanations, setting goals, making choices, and taking action (Zimbardo and Boyd, 2015). Among them, the time tendency of present hedonistic will make individuals more immersed in mobile phone addiction and other behavioral addictions or substance abuse. Zimbardo and Boyd (1999) proposed the balanced time perspective (BTP) concept made up of a specific combination of the five temporal cognitive dispositions; more past positive, moderate present hedonic and future orientations, and less past negative and present fatalistic orientations (Zimbardo and Boyd, 1999; Zimbardo, 2002; Witowska and Zajenkowski, 2021). People with low DBTP can flexibly and appropriately shift between the three temporal domains based on task characteristics, environmental demands, and personal resources (Zimbardo and Boniwell, 2004).

Addiction research has found that past negative, present hedonic, and present fatalistic temporal perspective factors can facilitate addictive behaviors and increase the likelihood of occurrence and that a future orientation could be a protective factor that decreases the likelihood of addictive behavior (Przepiorka and Blachnio, 2016). When subjected to stressful events, people may develop DBTP, that is, they may overemphasize one time period (past, present, or future) and ignore others, which can have negative consequences (Zimbardo and Boniwell, 2004). For example, Gruber et al. (2012) found that mood disorders could often result in a greater focus on the present and neglect the future. The COVID-19 pandemic conditions, such as isolation and the lack of face-to-face social contact, saw an increase in negative emotions and disruptions to people’s temporal perspectives (Killgore et al., 2020; Tull et al., 2020), which in turn resulted in adverse behaviors, such as impulsivity and addiction (Lv and Huang, 2005).

Expectancy-Value Theory claims that outcomes, expectancy, and valuation drive people’s behavioral motivations and that future-oriented temporal insight can result in better regulation of current behaviors (Pang et al., 2014). Zimbardo’s time theory also claims that having a future-time perspective can assist people in leading successful lives and achieve happiness. Earlier research also suggested that having a future-time perspective could negatively impact student mobile addiction, as it was found that mobile addiction was low if the students’ time values were future-oriented (Park et al., 2014). People with low DBTP could shift flexibly between the three temporal categories and maintain a temporal perspective (Zimbardo, 2002).

*H1*: It is hypothesized that DBTP positively predicted adolescent mobile phone addiction during the COVID-19 epidemic.

### 1.2 The mediating role of depression and anxiety symptoms

Five temporal perspective factors have been associated with psychological well-being. An aversion to the past and fatalistic attitudes toward life are significantly and positively associated with trait anxiety and depression, having a future time perspective is positively associated with positive coping styles, and positive attitudes toward the past are negatively associated with depression and trait anxiety (Anagnostopoulos and Griva, 2012). A BTP, therefore, can help people better resolve problems and achieve higher well-being and mental health (Zimbardo and Boniwell, 2004; Griffin and Wildbur, 2020). However, DBTP can result in poorer well-being (Boniwell et al., 2010) and health (Oyanadel and Buela-Casal, 2011) and give rise to increased negative emotions, such as depression and anxiety. The cognitive-behavioral model of pathological internet use suggests that psychopathogens, such as depression and anxiety, can lead to pathological internet use behavioral symptoms (Davis, 2001). Many studies have found a relationship between mobile phone addiction, depression, and anxiety (Elhai et al., 2018). For example, Choksi and Patel (2021) found that stress, anxiety, and depression were significantly and positively associated with mobile phone addiction, Carli et al. (2013) found that depression, anxiety, and stress could lead to internet addiction, and Wang et al., 2020) found that psychological distress, such as depression and anxiety, mediated the relationship between academic stress and mobile phone addiction in adolescents.

*H2*: It is hypothesized that depression and anxiety played a mediating role in balancing the DBTP effects on mobile phone addiction.

### 1.3 The moderating role of self-control

Self-control, a stable personality trait, is the ability to regulate or control impulsive thoughts, feelings, and behavior (Telzer et al., 2011) and may help regulate adverse mental health consequences from events such as a pandemic (Li et al., 2016). Because people with low self-control are more likely to be diagnosed with depression and other psychiatric disorders (Miller et al., 2011), good self-control could result in less incidence of adolescent depression (Dishion and Connell, 2006). Tangney et al. (2004) examined the relationship between self-control and key psychological symptoms using self-reports on psychopathological symptoms, such as anxiety, depression, obsessive compulsion, and somatic symptoms, and found that the respondents with low self-control were less psychologically resilient. The self-control power model (Baumeister et al., 2007) suggests that as people have limited self-control resources, when their psychological resources become depleted, there is a failure in the tasks that require self-control (Baumeister et al., 1998).

During the COVID-19 outbreak, many people used up their psychological resources to deal with the associated negative emotions and stress. Therefore, for people with low self-control, the BTP that regulates negative past or future emotions may have failed, which could have increased their likelihood of developing depressive and anxiety symptoms.

Mobile phone addiction is related to self-control (Lemola et al., 2015). The dual systems self-control model (Hofmann et al., 2009) claims that people with high self-control are more likely to be influenced by a reflective system that helps them delay gratification when they encounter negative life events; however, for people with low self-control, the impulsive system is engaged in intuitive heuristic processing to satisfy their current needs, which increases their tendency toward impulsive behavior, such as mobile phone addiction (Hofmann et al., 2009; Lu et al., 2019). Therefore, people with low self-control have greater difficulty resisting impulses, and are more likely to satisfy their urge to use their mobile phones to seek immediate comfort, eliminate stress and negative emotions from events such as the COVID-19 pandemic. That is, depressive anxiety is more likely to result in addictive mobile phone behaviors in people with lower self-control.

*H3*: Self-control plays a moderating role in balancing the second half of the DBTP effect pathway to mobile phone addiction.

**Figure 1:**
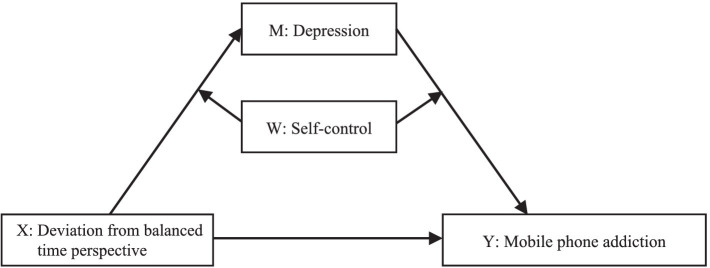
Model assumptions (I).

**Figure 2:**
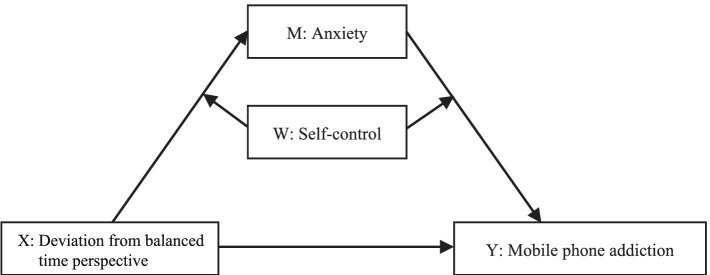
Model assumptions (II).

In summary, this study developed a mediated model that had depression and anxiety symptoms as the mediating variables and self-control as the moderating variable (Figures 1, 2). The role depression, anxiety, and self-control had on the DBTP effects on mobile phone addiction was systematically explored using empirical data. Specifically, whether depression and anxiety mediated the relationship between DBTP and mobile phone addiction and whether self-control moderated this mediation process was examined in the COVID-19 pandemic context.

## 2 Methods

### 2.1 Participants

Amidst the extensive COVID-19 outbreak, varying control policies were noted across distinct regions of China. To delve into this phenomenon, we systematically chose five out of 20 vocational schools in close proximity to Chengdu, Sichuan Province. A total of 1,300 online questionnaires were distributed across the selected schools, with approximately 230–300 questionnaires administered in each school. To protect subjects’ privacy, we built our platform to collect data under the project funding.

Stringent criteria were established to identify and exclude invalid samples. Such criteria included completion times deemed excessively short and a notable number of unanswered items, defined as more than five questions left unattended. Adhering to these criteria, a total of 136 questionnaires were deemed invalid and subsequently excluded from the analysis. Finally, after eliminating invalid responses, 1,164 valid questionnaires (324 males, 840 females) were returned, an effective return rate of 89.53% (Table 1). The average age was 18.26 ± 1.50 years old and most had parents with only junior high school or below. All participants signed an informed consent form before completing the questionnaire, and all procedures in this study met the ethical standards of the research committee of Sichuan University.

**Table 1:** Demographic participant information (*N* = 1,164).

Variables	Classification	Number	Proportion (%)
Gender	Male	324	27.8
	Female	840	72.2
Age	15	101	8.7
	16	95	8.2
	17	38	3.3
	18	366	31.4
	19	294	25.3
	20	270	23.2
Father’s education level	Below junior high school	766	65.8
	High School	242	20.8
	Specialties	105	9.0
	Undergraduate	49	4.2
	Postgraduate students	2	0.2
Mother’s education level	Below junior high school	847	72.8
	High School	207	17.8
	Specialties	71	6.1
	Undergraduate	35	3.0
	Postgraduate students	4	0.3
Primary Guardian	1 Parents	663	57.0
	2 Father	38	3.3
	3 Mother	127	10.9
	4 Grandparents	204	17.5
	5 Residential	132	11.3

### 2.2 Materials

#### 2.2.1 Basic personal information

The basic personal information questionnaire gathered basic demographic data: gender, age, father’s education level, mother’s education level, and primary guardian.

#### 2.2.2 Deviation from balanced time perspective

The Zimbardo time perspective inventory (ZTPI) (Zimbardo and boyd, 1999) was used, which has 19 items across five dimensions; positive past, negative past, fatalistic present, hedonistic present, and future orientation; which are scored on a five-point scale from 1 = extremely unlikely to 5 = extremely consistent based on the participant’s feelings about the question content. Example questions are: It does not really matter what I do since things are going to happen in the future, and to achieve my dreams, I will set goals and consider concrete steps to achieve them. The score for each dimension was the average of the total score for each dimension divided by the number of questions in that dimension, with higher scores indicating higher BTP. Cronbach’s alpha was 0.905 for this measure. The DBTP method in Stolarski et al. (2011) was used to determine the BTP, which employed the following formula:


$$\mathrm{DBTP}=\sqrt{\begin{array}{l} {\left(oPN-ePN\right)}^{2}+{\left(oPP-ePP\right)}^{2}+{\left(oPF-ePF\right)}^{2}+\\ {\left(oPH-ePH\right)}^{2}+{\left(\mathrm{oF}-eF\right)}^{2} \end{array}}$$

where oPN, oPP, oPF, oPH, and oF were the respective scores for the past negative, past positive, present fatalistic, present hedonic, and future, and ePN, ePP, ePF, ePH, and eF were the optimal critical values for each dimension. The method calculates the difference between the actual and ideal scores on the five dimensions, squares the five differences and adds them together, and then finally squares the sum of the squares for the five differences. The optimal critical value was also revised to fit the Chinese version of ZTPI as reported in Jankowski et al. (2020), Li et al. (2022) and Li and Lv (2022) the higher the DBTP, the more unbalanced the DBTP.

#### 2.2.3 Depression and anxiety symptoms

The brief symptom inventory of physical and mental health (BSI-18) was used, which is an 18-item questionnaire with three dimensions; somatization, depression, and anxiety; and includes depression questions focused on “feeling lonely” and “not interested in things,” anxiety questions asking about “nervousness and insecurity” or “feeling nervous or easily stressed,” which were scored on a 0–4 scale from never to very severe; the higher the total score, the poorer the participant’s recent mental health. In the sample under examination, the BSI demonstrated robust internal consistency, with Cronbach’s alpha coefficients of 0.91 for depression and 0.92 for anxiety. Comparatively, recent literature indicates that reliability coefficients for depression range from 0.84 to 0.91, as reported by Geng et al. (2022), and for anxiety, they range from 0.86 to 0.91, as noted by Li et al. (2018). The broader reliability and validity range across studies is reported to be between 0.87 and 0.92, according to Zhang et al. (2022a). These findings suggest that the utilization of the BSI in the present research aligns well with established standards in the literature, reinforcing its credibility as a reliable and valid instrument for assessing depression and anxiety.

#### 2.2.4 Mobile phone addiction

The Chinese version of the mobile phone addiction index (MPAI) scale developed by Leung (2008) and translated by Huang et al. was also used. The scale, which is suitable for adolescent use, has 17 items over four dimensions; loss of control, withdrawal, avoidance, and ineffectiveness; and was scored on a five-point scale from 1 = very unlikely to 5 = very likely. Sample questions were; “Ever lied to family, friends, or others to hide the amount of time you spend on your phone” and “You spend more time on your phone than you planned.” The total score was a summation of all items, with higher scores indicating higher mobile phone addiction. Participants were classified as mobile phone addicts if they responded positively to eight out of the 17 items. Cronbach’s alpha coefficient for this measure was 0.90.

#### 2.2.5 Self-control

The self-control scale (SCS) revised by Shuhua and Guo was used, which has 19-items over five dimensions; impulse control, healthy habits, resisting temptation, focusing on work, and abstaining from recreation; and is scored on a five-point scale from 1 = not at all to 5 = very in line. Sample questions were; “I can resist temptation well” and “It is difficult for me to change bad habits.” The total self-control score was the sum of the scores for each topic, with the higher the score, the better the self-control. Cronbach’s alpha coefficient for this measurement was 0.77.

### 2.3 Study procedures and data processing

The questionnaires were distributed online between February and March 2020, the period of the first COVID -19 outbreak in China, at which time all students were isolated at home for more than 2 months. The survey instruments were independently completed, and after the questionnaires were returned, the data were entered and verified using SPSS 20.0, which was also used for the statistical analysis. Model 58 in the PROCESS macro (Hayes, 2018) (downloaded from^1^) was used to test the moderating and mediating effects. Standard errors (SE) and 95% confidence intervals (95% CI) were obtained for the parameter estimates, with the statistical results indicating significance if the 95% confidence interval did not contain zero.

As the data were from self-reports, common method bias may have existed. Therefore, the Harman one-way test was used to examine if there were any common method bias effects (Podsakoff et al., 2012). The Harman one-way test showed that there were 21 factors with eigenvalues greater than 1 with the variance explained by the first factor being 17.80%, which was less than the critical 40% value; therefore, no serious common method bias was found.

## 3 Results

### 3.1 Correlations between DBTP, depression and anxiety, self-control, and mobile phone addiction

The results (see Table 2) showed that there was a significant positive correlation between DBTP, depression and anxiety, self-control, and mobile phone addiction (*r* = 0.415, *p* < 0.01), anxiety (*r* = 0.398, *p* < 0.01) and self-control (*r* = −0.508, *p* < 0.01) and mobile phone addiction (*r* = 0.475, *p* < 0.01), depression and self-control (*r* = −0.333, *p* < 0.01), anxiety and self-control (*r* = −0.322, *p* < 0.01), self-control and mobile phone addiction (*r* = −0.585, *p* < 0.01), and self-control and mobile phone addiction (*r* = − 0.585, *p* < 0.01).

**Table 2:** Descriptive statistics and Pearson’s correlation analysis (*N* = 1,164).

	1	2	3	4	5	6	7	8	9	10
1. Gender	–	0.168^**^	−0.025	−0.048	−0.002	0.028	0.055	0.056	−0.107^**^	0.052
2. Age		–	0.078^**^	0.050	0.043	0.001	0.011	−0.040	0.048	−0.055
3. Father’s education level			–	0.576^**^	−0.067^*^	0.064^*^	0.046	0.015	−0.044	0.017
4. Mother’s education level				–	−0.015	0.089^**^	0.022	−0.010	−0.028	0.026
5. Primary Guardian					–	0.094^**^	0.089^**^	0.067^*^	−0.100^**^	0.045
6. DBTP						–	0.415^**^	0.398^**^	−0.508^**^	0.475^**^
7. Depression							–	0.845^**^	−0.333^**^	0.269^**^
8. Anxiety								–	−0.322^**^	0.267^**^
9. Self-control									–	−0.585^**^
10. Mobile phone addiction										–

Gender: 1 = Male; 2 = Female.

Parental education level: 1 = “below junior high school,” 2 = “high school,” 3 = “specialist,” 4 = “undergraduate,” 5 = “Postgraduate”; Primary guardian: 1 = “parent,” 2 = “father,” 3 = “mother,” 4 = “grandparents,” 5 = “living at school”.

^*^p < 0.05, ^**^p < 0.01.

### 3.2 The moderated mediation model

Firstly, this study analyzed the simple mediating effects of depression and anxiety, in which gender, father’s education, mother’s education, primary guardian, and age were controlled for. The results show that, DBTP was positively associated with mobile phone addiction (*β* = 0.474, *p* < 0.001). And, When the mediating variable is depression, the direct effect was significant (*β* = 0.439, *p* < 0.001); When it’s anxiety, the direct effect was also significant (*β* = 0.440, *p* < 0.001). Furthermore, the indirect effect of DBTP on mobile phone addiction through depression was significant (*β* = 0.035, SE = 0.013, 95% CI = [0.010, 0.062]), through anxiety was also significant (*β* = 0.034, SE = 0.013, 95% CI = [0.010, 0.061]). Therefore, depression and anxiety mediated the effect of DBTP on mobile phone addiction.

A moderating mediator model with anxiety and depression as the mediating variables and self-control as the moderating variable was constructed. The above additional variables were still controlled. The results are presented in Tables 3, 4.

**Table 3:** Mediated model (I) with moderation (*N* = 1,164).

Predictor variables	Depression			Mobile phone addiction			
	*β*	*t*	95% CI	*β*	*t*	95% CI	
Gender	0.061	1.025	[−0.056, 0.177]	−0.004	−0.069	[−0.107, 0.099]	
Age	0.008	0.449	[−0.027, 0.043]	−0.021	−1.309	[−0.051, 0.010]	
Father’s education level	0.052	1.362	[−0.023, 0.127]	−0.027	−0.804	[−0.093, 0.039]	
Mother’s education level	−0.058	−1.399	[−0.140, 0.024]	0.008	0.222	[−0.064, 0.081]	
Primary Guardian	0.029	1.689	[−0.005, 0.063]	−0.016	−1.075	[−0.046, 0.014]	
DBTP	0.327	10.789^***^	[0.268, 0.387]	0.232	8.257^***^	[0.177, 0.288]	
Self-control	−0.147	−4.799^***^	[−0.207, −0.087]	−0.463	−16.945^***^	[−0.516, −0.409]	
DBTP ×self-control	−0.126	−6.267^***^	[−0.165, −0.087]				
Depression				0.058	2.018^*^	[0.002, 0.115]	
Depression × self-control				0.055	2.748^**^	[0.016, 0.094]	
*R*	0.472			0.625			
*R*^2^	0.223			0.391			
*F*	41.320^***^			82.367^***^			

^***^p < 0.001, ^**^p < 0.01, ^*^p < 0.05.

**Table 4:** Analysis of mediated model (II) with moderation (*N* = 1,164).

Predictor variables	Anxiety			Mobile phone addiction		
	*β*	*t*	95% CI	*β*	*t*	95% CI
Gender	0.081	1.358	[−0.036, 0.198]	−0.009	−0.162	[−0.112, 0.095]
Age	−0.025	−1.424	[−0.060, 0.010]	−0.020	−1.271	[−0.051, 0.011]
Father’s education level	0.031	0.801	[−0.045, 0.106]	−0.026	−0.786	[−0.093, 0.040]
Mother’s education level	−0.082	−1.950	[−0.165, 0.001]	0.011	0.304	[−0.061, 0.084]
Primary Guardian	0.016	0.923	[−0.018, 0.050]	−0.015	−1.012	[−0.045, 0.014]
DBTP	0.317	10.388	[0.257, 0.377]	0.232	8.290^***^	[0.177, 0.287]
Self-control	−0.140	−4.560	[−0.201, −0.080]	−0.464	−17.016^***^	[−0.517, −0.410]
DBTP × self-control	−0.130	−6.400	[−0.169, −0.090]			
Anxiety				0.064	2.282^*^	[0.009, 0.119]
Anxiety × self-control				0.063	3.149^**^	[0.024, 0.101]
*R*	0.459			0.627		
*R*^2^	0.210			0.393		
*F*	38.479^***^			82.854^***^		

^***^p < 0.001, ^**^p < 0.01, ^*^p < 0.05.

The moderated mediated model found that DBTP positively predicted mobile phone addiction. The moderated mediation model was tested using the PROCESS model 58, and after controlling for gender, age, father’s education, mother’s education, and primary guardian, the results (see Table 3) indicated that the interaction between DBTP and self-control in the model hypothesis one was significant (*β* = −0.126, *p* < 0.001) as was the interaction between depression and self-control (*β* = 0.055, *p* < 0.01). The interaction between DBTP and self-control in model hypothesis two was also significant (*β* = −0.130, *p* < 0.001), as was the interaction between anxiety and self-control (*β* = 0.063, *p* < 0.01). Therefore, self-control moderated the entire indirect pathway, which supported the study hypothesis 3.

According to the mean ± 1 standard deviation, independent self-construal was divided into high (M + 1SD) and low (M-1SD) groups, Scale score from low independent self-construal (M-1SD = 52.98) to high independent self-construal (M + 1SD = 70.54). The predictive effect of DBTP on mobile phone addiction at different self-control levels was further investigated using simple slope analysis.

**Figure 3:**
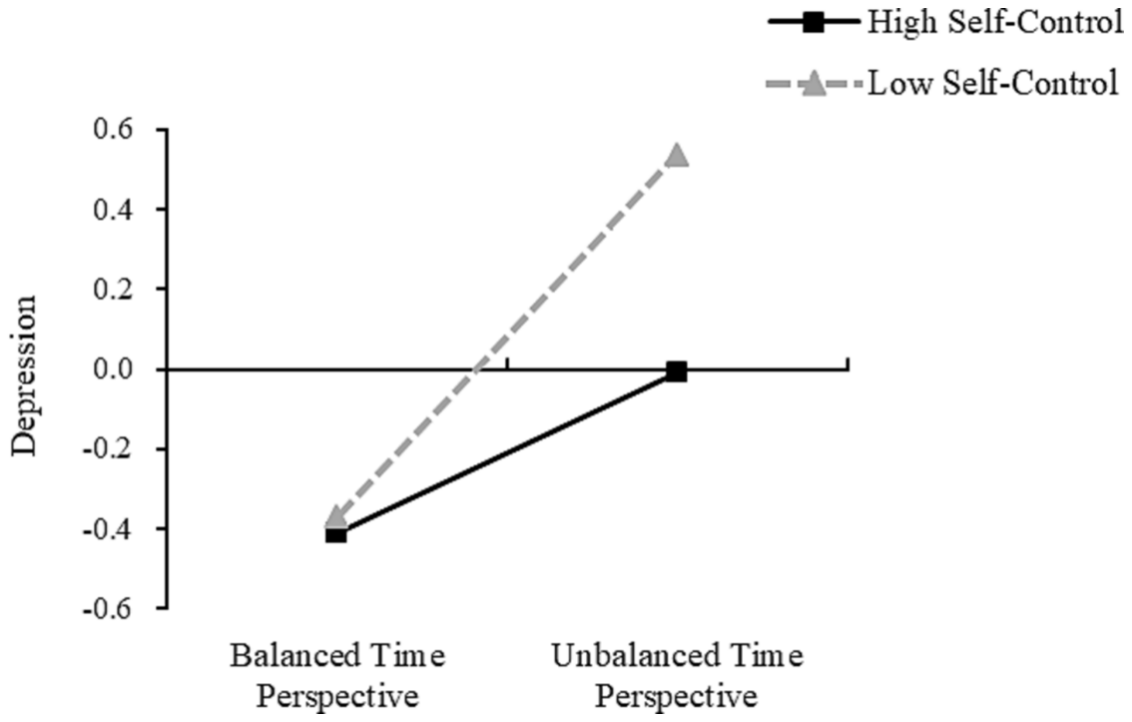
Moderating role of self-control in balancing the effects of DBTP on depression.

**Figure 4:**
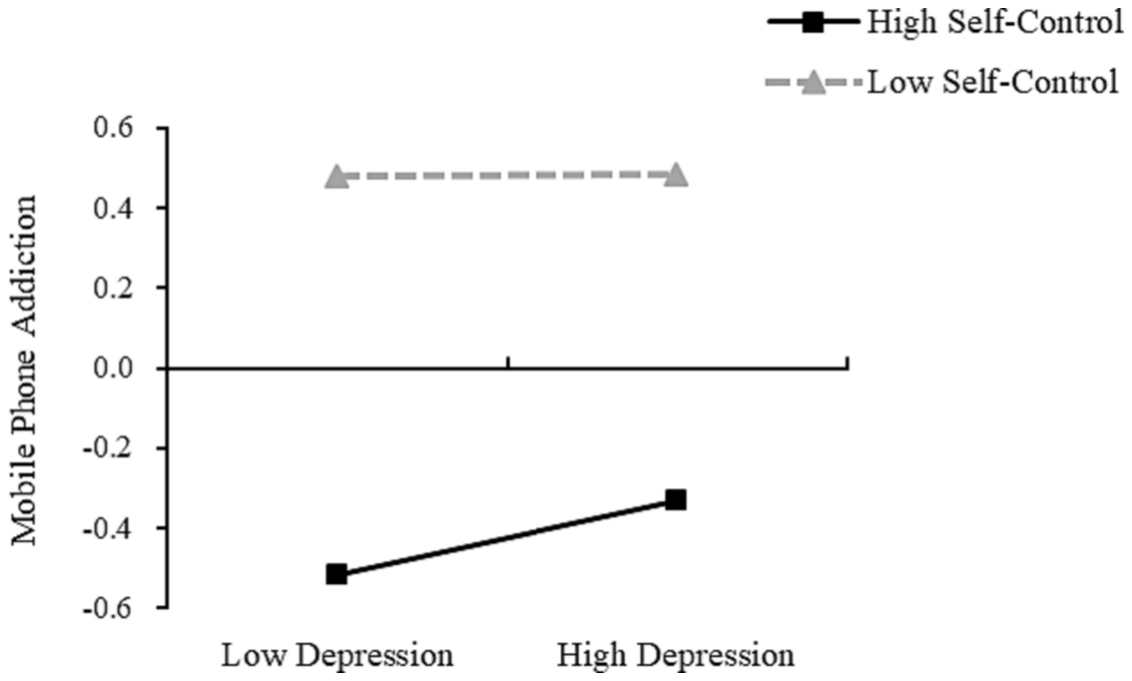
Moderating role of self-control on the influence of depression on mobile phone addiction.

It was found that in Model 1, DBTP significantly predicted depression when the self-control was low, with the simple slope being; *b* = 0.453 (*t* = 12.550, *p* < 0.001, SE = 0.036, 95%CI [0.382, 0.524]). DBTP also significantly predicted depression when self-control was high, with the simple slope being; *b* = 0.201 (*t* = 5.492, *p* < 0.001, SE = 0.037, 95%CI [0.129, 0.273]) (Figure 3). At the same time, when self-control levels were low, depression was not a significant predictor of mobile phone addiction, with the simple slope being; *b* = *0*.003 (*t* = 1.293, *p* = 0.897, SE = 0.027, 95%CI [−0.049, 0.055]). However, as self-control increased, depression was found to be a significant predictor of mobile phone addiction, with the simple slope being; *b* = 0.113 (*t* = 2.700, *p* < 0.01, SE = 0.042, 95%CI [0.031, 0.195]) (Figure 4). Further, the indirect effect size of depression was 0.002 (Boot SE = 0.015, 95% CI [−0.029, 0.030]), and the mediating effect was not significant. At high levels of self-control, the indirect effect size of depression was 0.023 (Boot SE = 0.011, 95% CI [0.004, 0.047]), and the mediating effect was significant, accounting for 65.71% (0.023/0.035) of the total indirect effect and 4.85% (0.023/0.474) of the total effect.

**Figure 5:**
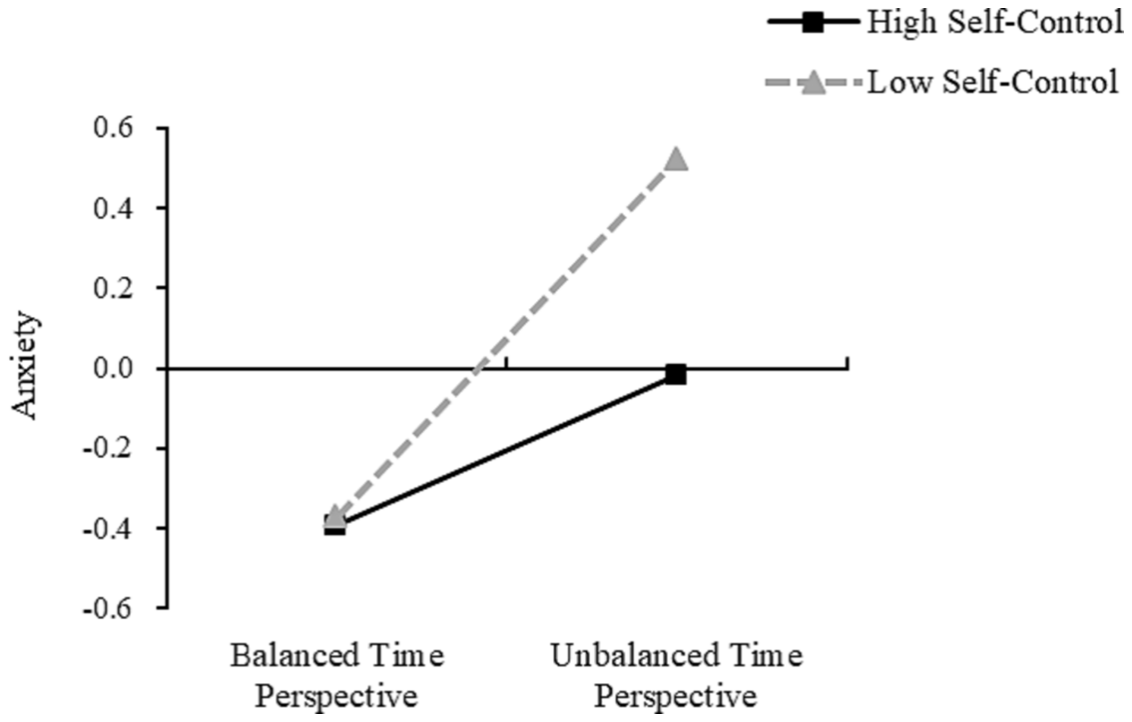
Moderating role of self-control in balancing the effects of DBTP on anxiety.

**Figure 6:**
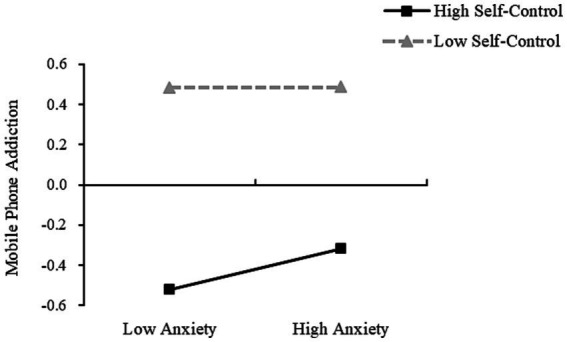
Moderating role of self-control on the influence of anxiety on mobile phone addiction.

In Model 2, the DBTP significantly predicted anxiety when self-control was low, with the simple slope being; *b* = 0.447 (*t* = 12.287, *p* < 0.001, SE = 0.036, 95%CI [0.376, 0.518]); and also when the self-control was high, with the simple slope being; simple slope *b* = 0.188 (*t* = 5.087, *p* < 0.001, SE = 0.037, 95%CI [0.115, 0.260]) (Figure 5). At low self-control, anxiety did not significantly predict mobile phone addiction, with the simple slope being; *b* = 0.002 (*t* = 0.060, *p* = 0.952, SE = 0.027, 95%CI [−0.051, 0.054]). As the self-control increased, anxiety was found to significantly predict mobile phone addiction, with the simple slope being; *b* = 0.127 (*t* = 3.108, *p* < 0.01, SE = 0.041, 95%CI [0.047, 0.206]) (Figure 6). In addition, at low levels of self-control, the indirect effect of anxiety is 0.001 (Boot SE = 0.016, 95% CI [−0.031, 0.031]), and the mediating effect is not significant; At a high level of self-control, the indirect effect of anxiety is 0.024 (Boot SE = 0.012, 95% CI [0.004, 0.053]), and the mediating effect is significant, accounting for 68.57% (0.024/0.035) of the total indirect effect and 5.06% (0.024/0.474) of the total effect. Table 5 shows the effect sizes of all model paths.

**Table 5:** Effect sizes of all model paths.

Model	Paths	Effect value (*β*)	Boot SE	95% confidence interval	effect ratio
Model 1: depression	Total effect	0.474	0.026	[0.423, 0.525]	–
	Direct effect	0.439	0.028	[0.383, 0.495]	92.62%
	Total indirect effect	0.035	0.013	[0.010, 0.062]	7.38%
	Low level moderator	0.002	0.015	[−0.029, 0.030]	–
	High level moderator	0.023	0.011	[0.004, 0.047]	4.85%
Model 2: anxiety	Total effect	0.474	0.026	[0.423, 0.525]	–
	Direct effect	0.440	0.028	[0.384, 0.495]	92.83%
	Total indirect effect	0.034	0.013	[0.010, 0.061]	7.17%
	Low level moderator	0.001	0.016	[−0.030, 0.031]	–
	High level moderator	0.024	0.012	[0.005, 0.052]	5.06%

## 4 Discussion

This study examined the impact of DBTP on mobile phone addiction and the underlying mechanisms in Chinese high school students during the COVID-19 epidemic. It was found that the more severe the DBTP, the higher the depression and anxiety and the higher the mobile phone addiction. It was also observed that self-control played an important moderating role in this process and was an important protective factor against the negative emotions arising from DBTP and mobile phone addiction.

The DBTP had a significant positive association with mobile phone addiction, which was consistent with previous research that the more unbalanced the DBTP, the higher the mobile phone addiction (Meng et al., 2021). When people are less time-sensitive, they tend to neglect time management and indulge in mobile phone use in the present, which can lead to excessive addiction (Yumeng et al., 2020). Focusing on the future and having a better insight into the future time can protect against mobile phone overuse triggered by negative states such as boredom (Yang et al., 2021). Przepiorka and Blachnio (2016) explored the relationship between different factors and internet addiction and found that hedonistic orientations had a significant positive predictive effect. Adolescents with better BTPs tend not to have a certain temporal orientation, are not overly influenced by negative pasts and the present, and are therefore not detached from unrealistic present and future expectations, that is, they have a greater capacity for negative state self-regulation. Therefore, during the COVID-19 outbreak, they were able to focus more on real-life experiences, perceive real life, and plan for their future, that is, these students restrained from seeking instant gratification from their mobile phones and the internet to relieve the associated pandemic stress and negative emotions. In summary, DBTP significantly predictes mobile phone addiction in adolescents.

Second, the findings suggested that depression and anxiety played a mediating role in the DBTP effect on mobile phone addiction, which was in line with previous research that mental health worsens when DBTP increases (Oyanadel and Buela-Casal, 2014), which for adolescents tends to lead to higher mobile phone addiction (Li et al., 2022). The COVID-19 pandemic resulted in an increase in mental health issues, such as anxiety, depression (Shanafelt et al., 2020), panic, and other negative emotions (Hagerty and Williams, 2020). Although the mediating effect size of anxiety and depression in our study was not large (7.38 and 7.17%), the significant results consistent with previous studies still indicate that negative psychological state plays a non-negligible role in the influence path of DBT on adolescent mobile phone addiction. People with BTPs were generally able to maintain positive self-focus and self-esteem and positive views about their self-worth. This strong sense of self-continuity over time allowed them to strengthen their relational bonds, and provided a sense of meaning and purposeful living. Therefore, these results indicated that adolescents with BTPs were less likely to experience depression and anxiety during the COVID-19 pandemic (Anagnostopoulos and Griva, 2012). In contrast, people with DBTP were more likely to be dissatisfied with their current life, believe life would not improve and the future was predetermined and not influenced by personal behavior. Therefore, young people with DBTP who lacked personal agency during the COVID-19 lockdowns were more likely to be trapped in anxiety and depression, with their reduced offline social interactions leading to increased mobile phone addiction (Ma et al., 2021).

An earlier psychopathological meta-analysis found that depression and anxiety severity were consistently associated with problematic mobile phone use (Elhai et al., 2017), and a Taiwan study found that adolescents with major depressive disorders were more likely to have four or more problematic mobile phone use symptoms (Yen et al., 2009). It has also been found that negative personal emotions, such as depression and anxiety, can influence mobile phone addiction in college students, especially when they are suffering from significant depression, anxiety, and/or stress (Gao et al., 2018). Elhai et al. (2019) also found that mobile phone overuse was associated with anxiety and depressive symptoms, and Kim et al. (2019) found that people with lower self-perceived emotional or health statuses were more likely to exhibit excessive mobile phone use to compensate for or overcome their emotional issues without realizing that this addiction contributed to their negative emotional and physical health. In summary, depression and anxiety play a mediating role in balancing the DBTP effect on mobile phone addiction.

Finally, the results suggested that self-control moderated the effect of DBTP on mobile phone addiction during the COVID-19 epidemic. Self-control moderated the first half of the DBTP pathway to mobile phone addiction through depression and anxiety, with the stronger the self-control, the lower the risk of DBTP leading to depression and anxiety. The limited self-control theory claims that self-control and mental health are closely related and that externally stressful situations can deplete self-control resources, which in turn can lead to maladjustment or emotional behavioral problems (Tan and Guo, 2008). Clinical studies have found that people with lower self-control are significantly more likely to be diagnosed with depression and other psychiatric disorders (Miller et al., 2011) and people with higher self-control have better inhibition and initiation (Li et al., 2016) and use more positive coping strategies and fewer negative coping strategies (Duckworth and Seligman, 2005). Therefore, during the COVID-19 epidemic, self-control was a protective factor against depressive anxiety and DBTP. Self-control was found to moderate the second half of the pathway through depression and anxiety to mobile phone addiction, with the stronger the self-control, the lower the risk of depression and anxiety and the lower the mobile phone addiction. The dual systems self-control model (Hofmann et al., 2009) claims that when adolescents have negative emotional states, such as the depression and anxiety experienced during the COVID-19 pandemic period or from DBTP, good self-control could result in greater behavioral reflection, which could have reduced or eliminated the stress and negative emotions from the pandemic and the need to seek emotional comfort through negative behaviors such as mobile phone addiction. Therefore, self-control can reduce the need to seek gratification when encountering negative life events and is a protective factor against the depressive anxiety that can result in addictive mobile phone behavior. In summary, self-control moderates the effects of DBTP on adolecent mobile phone addiction during the COVID-19 pandemic.

Although the impact of COVID-19 on people’s lives has diminished, recent research suggests that the interplay between variables such as self-control, anxiety, and time perception continues to significantly influence adolescent smartphone addiction (Liu et al., 2022; Peng et al., 2022; Zhang et al., 2022b). The prevalence of smartphone addiction among adolescents persists beyond the resolution of pandemic-related restrictions. These findings align with the conclusions drawn in this research, emphasizing the enduring relevance of adolescent smartphone addiction dynamics observed during the COVID-19 period in the post-pandemic real-world context.

The assumed variable relationship model in this study is grounded in prior relevant research and theories, extending back to before 2019, as introduced in the preamble. The study does not assert the exclusivity of the model to the context of COVID-19. Taking a broader temporal perspective, COVID-19 represents just one among various stressors individuals encounter in their daily lives. In the spectrum of stress events, COVID-19 does not possess uniquely significant characteristics. Consequently, the study’s conclusions drawn from the COVID-19 period can be extrapolated to more general contexts, encompassing a range of stress events and not limited to the specific circumstances of the pandemic.

This research focuses on increased psychological distress and smartphone addiction among high school students during the COVID-19 pandemic. It posits that Behavioral Tendency to Perseveration (BTP) is associated with these changes and that individuals with higher BTP are more prone to psychological distress and smartphone addiction compared to those with lower Defensive Behavioral Tendency to Perseveration (DBTP). Although BTP is commonly considered a stable individual difference (Jochemczyk et al., 2017), in the context of collective trauma like COVID-19, individual BTP may deviate, leading to more significant psychological distress (Holman and Grisham, 2020). Consequently, the relationship between DBTP and smartphone addiction persists beyond the disappearance of COVID-19, becoming more evident in stressful situations such as the pandemic, where a higher prevalence of DBTP is observed.

Simultaneously, this study explores the role of self-control as a protective factor against adolescent behavioral issues, demonstrating a significant impact regardless of whether the context involves COVID-19 (Li et al., 2021). According to the self-control power model (Baumeister et al., 2007), there might be a reduction in self-control during the COVID-19 period, potentially diminishing its protective effects. However, the findings of this research indicate that self-control remains meaningful even during the pandemic, emphasizing its crucial role both in normal circumstances and during public health events like COVID-19. This underscores the applicability of the study’s conclusions to the current post-pandemic era.

## 5 Limitations and future studies

First, the cross-sectional design of the study did not allow for causal inferences to be made; therefore, future studies should use longitudinal or experimental designs to confirm the causal relationships between these variables. Second, the self-report methods may have resulted in social desirability and other biases that could limit data validity. Therefore, multiple assessments (adolescents, peers, parents, and teachers) could be used in future studies to provide more reliable evidence. Third, past research on mobile phone addiction was mainly conducted at the behavioral level and did not involve an exploration of the mobile phone addiction hindbrain mechanisms. Future research could therefore extend this study by exploring the neurophysiological mobile phone addiction mechanisms using modern equipment, such as functional magnetic resonance, near-infrared, and electroencephalography, which could provide reliable electrophysiological evidence for mobile phone addiction research and intervention.

Although the observed indirect effect sizes in our study were modest (7.38 and 7.17%), their statistical significance (*p* < 0.05) implies that this outcome is unlikely to be attributed to chance variation. It is crucial to acknowledge that, in certain contexts, even minor effect sizes can bear practical significance. In the context of our research, marginal improvements, when aggregated at the group level within the substantial target population, may exert a positive influence on guiding interventions for adolescent mental health and addressing mobile phone addiction. Nevertheless, it is essential to recognize that, given the magnitude of the effect, these changes might be less discernible at the individual level. Future investigations should contemplate the utilization of more sophisticated measurement tools or an extended evaluation period to comprehensively grasp the potential ramifications of this phenomenon. Simultaneously, the sizable sample size employed in our study may enhance the likelihood of detecting small effect sizes, underscoring the need for caution when interpreting these statistically significant results. Encouragement is extended for subsequent studies to delve deeper into the practical application and enduring effects of these preliminary findings.

## Data availability statement

The raw data supporting the conclusions of this article will be made available by the authors, without undue reservation.

## Ethics statement

The studies involving humans were approved by the Ethics Committee of Sichuan University. The studies were conducted in accordance with the local legislation and institutional requirements. Written informed consent for participation in this study was provided by the participants’ legal guardians/next of kin.

## Author contributions

HZ: Data curation, Investigation, Methodology, Writing – original draft. CC: Investigation, Methodology, Writing – review & editing. LZ: Supervision, Writing – review & editing. SX: Writing – review & editing, Methodology. WT: Supervision, Writing – review & editing, Data curation, Investigation.

## Footnotes

^1^http://www.Afhayes.com
